# Venomics of the milos viper (*Macrovipera schweizeri*) unveils patterns of venom composition and exochemistry across blunt-nosed viper venoms

**DOI:** 10.3389/fmolb.2023.1254058

**Published:** 2023-08-31

**Authors:** Lennart Schulte, Maik Damm, Ignazio Avella, Lilien Uhrig, Pelin Erkoc, Susanne Schiffmann, Robert Fürst, Thomas Timm, Günter Lochnit, Andreas Vilcinskas, Tim Lüddecke

**Affiliations:** ^1^ Department of Bioresources, Fraunhofer Institute for Molecular Biology and Applied Ecology, Giessen, Germany; ^2^ Institute for Insect Biotechnology, Justus Liebig University Giessen, Giessen, Germany; ^3^ LOEWE-Centre for Translational Biodiversity Genomics, Frankfurt, Germany; ^4^ CIBIO, Research Centre in Biodiversity and Genetic Resources, InBIO Associated Laboratory, University Port, Porto, Portugal; ^5^ Department of Biology, Faculty of Sciences, University of Porto, Porto, Portugal; ^6^ CIBIO, BIOPOLIS Program in Genomics, Biodiversity and Land Planning, Vairão, Portugal; ^7^ Institute of Pharmaceutical Biology, Faculty of Biochemistry, Chemistry and Pharmacy, Goethe University Frankfurt, Frankfurt, Germany; ^8^ Fraunhofer Institute for Translational Medicine and Pharmacology (ITMP), Frankfurt, Germany; ^9^ Institute of Biochemistry, Justus Liebig University Giessen, Giessen, Germany

**Keywords:** blunt-nosed vipers, venom, proteomics, bioactivity, snakebite

## Abstract

**Introduction:** Snakebite is a neglected tropical disease and a globally important driver of death and morbidity. Vipers of the genus *Macrovipera* (Viperidae: Viperinae) are among the snakes of higher medical importance in the Old World. Despite the medical relevance of *Macrovipera* venoms, the knowledge regarding them is heterogeneously distributed with virtually all works conducted so far focusing on subspecies of *Macrovipera lebetinus*, while other species within the genus are largely overlooked. Here we present the first proteomic evaluation of the venom from the Greek endemic Milos viper (*Macrovipera schweizeri*). In line with clinical symptoms typically elicited by *Macrovipera* envenomations, Milos viper venom primarily comprises coagulotoxic and cytotoxic protein families, such as metalloproteinases (svMP) and serine proteases (svSP).

**Methods:** We conducted comparative bioactivity assays on venoms from *M. schweizeri* and the *M. lebetinus* subspecies *M. lebetinus cernovi*, *M. lebetinus obtusa*, and *M. lebetinus turanica*, and showed that they all exhibit similarities in levels of cytotoxicity proteolytic activity, and inhibition of prokaryotic growth. Lastly, we compared *Macrovipera* venom profiles by 1D-SDS-PAGE and RP-HPLC, as well as our proteomic data with previously published *Macrovipera* venom proteomes.

**Results and discussion:** The analyzes performed to reveal that a general venom profile seems to be conserved across blunt-nosed vipers, and that, *M. schweizeri* envenomations, similarly to those caused by other blunt-nosed vipers, are able to cause significant tissue damage. The present work represents an important starting point for the development of comparative studies across the full taxonomic range of the genus *Macrovipera* and can potentially help optimize the treatment of envenomations caused by *M. schweizeri*.

## 1 Introduction

Snake venoms are complex cocktails of bioactive compounds (Calvete, 2013; Casewell et al., 2013), able to disrupt the physiological processes of the envenomated target (Fry et al., 2009). As a consequence of both, the evolutionary histories of divergent lineages and selection on the deployment of specific toxins, compositions and activities of snake venoms display extreme levels of variation, occuring at all taxonomic levels (Jackson et al., 2016; Tasoulis and Isbister, 2017; Casewell et al., 2020).

The evidence gathered so far suggests that one of the main factors contributing to the dynamic scenario of snake venom variation is adaptation to diet (Daltry et al., 1996; Casewell et al., 2020; Holding et al., 2021). In fact, assuming prey subjugation as the primary function of snake venom, its composition and activities are most likely shaped by strong natural selection in response to trophic factors such as prey availability, -preference, and/or -susceptibility to venom (Daltry et al., 1996; Casewell et al., 2013; Casewell et al., 2020). In this perspective, considering the adaptive value and the fast evolutionary rates of snake venom (Casewell et al., 2011), the occurrence of venom variation is most likely attributable to differences in diet and/or foraging strategies in snakes and beyond (Creer et al., 2003; Diniz et al., 2018; Mackessy et al., 2018; Casewell et al., 2020; Lüddecke et al., 2020; Fernandes et al., 2022). Studies detecting increased prey-specific lethality to natural prey appear to support the correlation between snake venom variation and snake feeding ecology types (e.g., Barlow et al., 2009; Gibbs and Mackessy, 2009). These findings are further backed by research developed on snake species adapted to prey types that do not require venom to be subdued where venom systems subsequently were lost or degenerated (e.g., Gopalakrishnakone and Kochva, 1990; Li et al., 2005).

The considerable attention that snake venom receives from researchers from all over the world is mainly attributable to the paramount medical relevance of snakebite (Kasturiratne et al., 2008; Gutiérrez et al., 2017; Roberts et al., 2022). Consistent with this, the venoms of viperid snakes are among the most studied ones most likely due to their critical impact on human health (Avella et al., 2022b). Indeed, pit vipers (subfamily Crotalinae) are the main cause of snakebite-related morbidity and mortality in the Neotropics (Gutiérrez, 2014; da Silva et al., 2019), while true vipers (subfamily Viperinae) are snakes of high medical importance in Africa, Asia and Europe (WHO, 2020). For instance, envenomations caused by blunt-nosed vipers (genus *Macrovipera*) are known to cause considerably severe, potentially lethal clinical manifestations in the Near and Middle East (Warrell, 2008; Amr et al., 2020; Dehghani et al., 2023), and are considered medically important also in Europe (Jestrzemski et al., 2022; Paolino et al., 2023).

**FIGURE 1:**
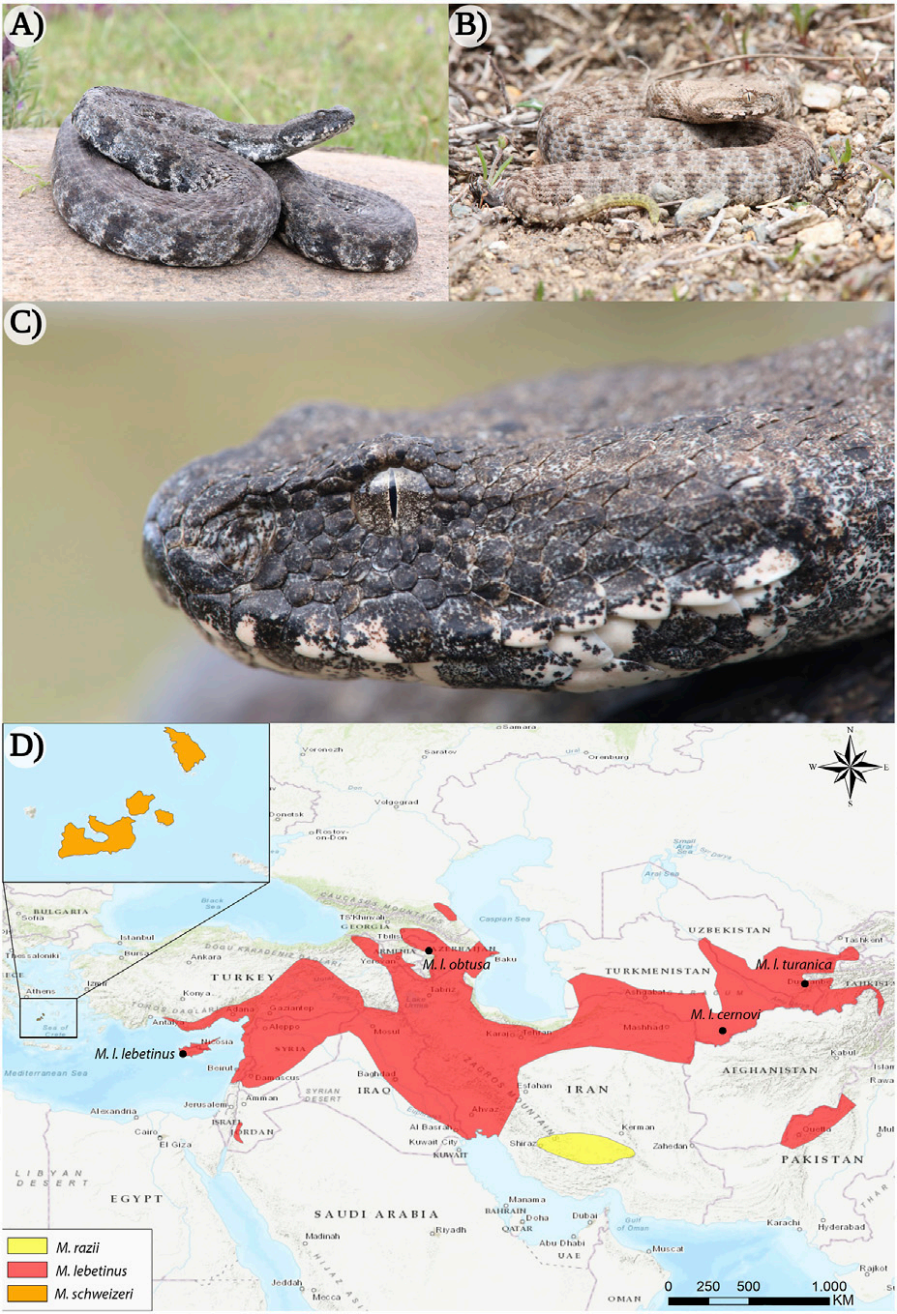
The appearance of the Milos viper (*Macrovipera schweizeri*) and distribution range of the genus *Macrovipera*. **(A)** Adult and **(B)** juvenile individuals of *M. schweizeri* from the island of Milos (Greece). **(C)** Close-up of the same adult animal depicted in **(A)**. **(D)** Distribution ranges of *Macrovipera* spp. (Böhme et al., 2009; Oraie et al., 2018; Aghasyan et al., 2021). Photo credits: Thomas Lindner.

Members of *Macrovipera* are large, thickset snakes, generally measuring about 100–150 cm in total length (Gruber, 1989; Baier et al., 2013; Geniez, 2018). Vipers of this genus occur on the islands on the Milos archipelago (Greece) and Cyprus, and range from southern Turkey to Tajikistan and northern Pakistan (Ananjeva et al., 2006; Sindaco et al., 2013; Speybroeck et al., 2016) (see Figure 1), typically inhabiting stony and semiarid habitats up to more than 2000 m of altitude (Oraie et al., 2018; Aghasyan et al., 2021). Although the systematic relationships within the genus are subject to controversial discussion among taxonomists (Stümpel and Joger, 2009; Freitas et al., 2020; Speybroeck et al., 2020). Three distinct species can be recognized within *Macrovipera*, namely the Levantine viper *Macrovipera lebetinus*, the Milos viper *Macrovipera schweizeri*, and Razi’s viper *Macrovipera razii*. Additionally, several subspecies have been described within *M. lebetinus*: *M. lebetinus lebetinus*, *M. lebetinus cernovi, M. lebetinus obtusa, M. l. transmediterranea* (often considered invalid Sindaco et al., 2013; Aghasyan et al., 2021) and *M. lebetinus turanica.*

The medical significance of *Macrovipera* spp. sparked an early and ongoing interest in elucidating compositions and activities of the venoms of these spectacular vipers, which appear to possess considerable value for drug research and development (Son et al., 2007; Park et al., 2009; Ozen et al., 2015). Considering their wide distribution (see Figure 1), blunt-nosed vipers are likely to experience different ecological and environmental conditions across their range, potentially resulting in different selective pressures. In the case that such pressures affect the vipers’ feeding ecology, it is reasonable to hypothesize that they may lead to venom variation. For instance, while *M. lebetinus* is reported to main feed on small mammals (Ščerbak and Bhöme, 2005), these constitute only a very small part of *M. schweizeri*´s diet, which appears to have adapted to almost exclusively feed on passerine birds (Nilson, 2018). Accordingly, *M. lebetinus* and *M. schweizeri*, exhibiting markedly different feeding ecologies but phylogenetically very close, appear as exploring the causes and the occurrence of venom variation. Interestingly, while recent studies focusing on *M. schweizeri* and *M. lebetinus* subspecies indicate potently procoagulant activity in all of them (Chowdhury et al., 2021a; Chowdhury et al., 2021b), as a consequence of the activation of blood coagulation factor X by snake venom metalloproteinase (svMPs) (Siigur et al., 2001; Chowdhury et al., 2021a). *Macrovipera schweizeri* venom appears to present the lowest factor X activation rates of the *Macrovipera* taxa examined (Chowdhury et al., 2021a). Furthermore, the taxon-specific neurotoxicity of various Palearctic vipers showed that, while the *M. lebetinus* subspecies *M. l. cernovi*, *M. l. obtusa* and *M. l. turanica* present a strong affinity for amphibian mimotopes, the venom of *M. schweizeri* targets more effectively lizard mimotopes (Chowdhury et al., 2022). Taken together, these results suggest the presence of interspecific venom variation within the genus *Macrovipera*.

Considering differences in feeding ecologies likely is one of are among the main drivers of snake venom variation, it is evident from the observations presented above that *M. schweizeri* potentially represents a key taxon to study this phenomenon in blunt-nosed vipers. However, while the venom compositions of several *M. lebetinus* subspecies were already investigated, virtually no data has so far been produced for the venom of *M. schweizeri*, despite its perhaps important role within the blunt-nosed viper venom variation conundrum. In the present study, we carry out the first qualitative assessment of the Milos viper venom composition, based on a shotgun proteomics approach. We pair our generated *M. schweizeri* venom proteome with electrophoretic and chromatographic profiling, bioactivity assays, and published *Macrovipera* venom proteomes, aiming to unveil potential compositional and functional differences within this genus. Our work aims to represent an important base for future comparative studies and argues for the importance of future quantitative investigations on the full taxonomic range of the genus *Macrovipera* to understand venom variation within blunt-nosed vipers.

## 2 Materials and methods

### 2.1 Venom samples

Following the decision of the Taxonomic Committee of the European Herpetological Society to maintain the Milos viper *M. schweizeri* at species level in the last update to the species list of the European herpetofauna (Speybroeck et al., 2020), in the present work we consider this taxon as species. Crude venoms of *M. schweizeri* (Greece) and the *M. lebetinus* subspecies *M. l. turanica* (Uzbekistan/ Turkmenistan), *M. l. obtusa* (Azerbaijan) and *M. l. cernovi* (Turkmenistan) were purchased from the venom supplier Latoxan (Portes-lès-Valence, France; https://www.latoxan.com/index.php, Table 1). The venoms were collected by milking their captive stock animals and were lyophilized before shipment. The obtained samples were stored at −20°C until further processing.

**TABLE 1:** Venom samples used in this study. Given are the taxa investigated herein, together with their country of origin and the respective product ID from their supplier (Latoxan).

Name	Origin	Product ID
*Macrovipera schweizeri*	Greece	L1127
*Macrovipera lebetinus cernovi*	Turkmenistan	L1144
*Macrovipera lebetinus obtusa*	Azerbaijan	L1126
*Macrovipera lebetinus turanica*	Uzbekistan, Turkmenistan	L1128

### 2.2 Venom proteomics

For the shotgun proteomic analysis, we used a mass spectrometry (MS) protocol previously used on different animal venoms (von Reumont et al., 2020; Hurka et al., 2022). Briefly, we dissolved 10 μg of sample material in 25 mM ammonium bicarbonate with 0.6 nM ProteasMaxTM (Promega). We added 5 mM DTT for 30 min at 50°C for disulfide reduction, followed by alkylation of free thiols via 10 mM iodacetamide for 30 min at 24°C. After quenching the reaction by excess cysteine, we added trypsin at a 50:1 ratio and digested the venom for 16 h at 37°C. After reaction stoppage by adding trifluoroacetic acid to a concentration of 1%, we purified the sample with C18-ZipTip (Millipore), dried them under a vacuum and redissolved the material in 10 μl of 0.1% trifluoroacetic acid.

Prior to mass spectrometry, we conducted a directly coupled chromatographic separation of the peptides on Thermo Fisher Scientific UltiMate 3000RSLCnano device (MA, USA). From the prepared sample material, we injected 1 μg into a 50 cm μPAC C18 column (Pharma Fluidics) in 0.1% formic acid at 35°C. Peptide elution was performed using a linear gradient of acetonitrile increasing from 3%–44% over 240 min at a flow rate of 300 nl/min. Finally, the column was washed with 72% acetonitrile. MS of the peptides was carried out on an Orbitrap Eclipse Tribrid MS (Thermo Fisher Scientific, MA, USA). Positive ionization with spray was established by an Advion TriVersa NanoMate (Advion BioSciences, NY, USA) with spray voltage set to 1.7 kV and source temperature set to 275°C. MS scans were performed in data-independent acquisition mode with the following settings: Scanning time 3 s, mass range of *m/z* 375-1,500 with resolution of 120,000. Auto-gain control was set to standard with a maximal injection time of 50 ms. The most intense ions occurring at each cycle with a threshold ion count of over 50,000 and charge states of 2-7 were selected with an isolation window of 1.6 m/z for higher-energy collisional dissociation (normalized collision energy 30%). Ion spectra of fragments were acquired in the linear ion trap with rapid scan rate and normal mass range. The maximum injection time was set to 100 ms and selected precursor ions were excluded for 15 s following fragmentation.

We used Xcalibur v4.3.73.11. (Thermo Fisher Scientific, MA, USA) and Proteome Discoverer v2.5.0.400 (Thermo Fisher Scientific, MA, USA) for data acquisition and analysis. Protein identification was performed with two different search engines and small peptide identities, such as tripeptides, were manual investigated and are listed separately within the supplementary information. Firstly in Mascot v2.8.2 (Matrix Science) searching against the UniProt database (taxonomy: “serpentes”) with following settings: Precursor ion mass tolerance of 10 ppm, carbamidomethylation as global modification, methionine oxidation as variable modification and one missed cleavage allowed. Fragment ion mass tolerance in linear ion trap MS2 detection was set to 0.8 Da and the false discovery rate was limited to 0.01 using a decoy database. For the qualitative analysis, we only considered proteins that were identified with a Mascot score of at least 30 and at least two verified peptides. As second proteome annotation PEAKS Studio 11.0 (build 20230414; Bioinformatics Solutions Inc., Canada), was carried out with the following settings: Parent Mass Error Tolerance (15.0 ppm), Fragment Mass Error Tolerance (0.5 Da), Precursor Mass Search Type (monoisotopic), Enzyme (Trypsin), Max Missed Cleavages (3), Digest Mode (Semi-Specific), Peptide Length Range (5–45). As post translational modifications (PTMs) carbamidomethylation (+57.02) was included as fixed and the following variable modifications: acetylation (K) (+42.01), HexNAcylation (N) (+203.08), hexose (NSY) (+162.05), oxidation (M) (+15.99), phosphorylation (STY) (+79.97), pyro-glu from E (−18.01), pyro-glu from Q (−17.03) and sodium adduct (+21.98) with a Max Variable PTM Per Peptide of 5. The database search was performed against 2,747 reviewed entries of Uniprot (taxonomy: “serpentes”; canonical and isoform; access 8th March 2023), including Deep Learning Boost (No) and FDR Estimation (Enabled). For the qualitative analysis, we only considered proteins that of a PEAKS score −10lgP (≥20), unique peptides ≥ 2 and a Peptide-Spectrum Matches (PSM) FDR of 0.01. A comprehensive list of all confidently identified venom components, their characteristics and annotation is given in Supplementary Tables S1–S4, proteomic raw data is available at PRIDE (PXD043615). The identified venom components were further grouped into major-, secondary, minor and rare venom components according to the classification system established by Damm et al., 2021. We counted the members of each protein family and manually calculated the relative abundances of the toxins families composing *M. schweizeri* venom. The calculation based upon the number identified venom proteins belonging to a single family (Supplementary Tables S2–S4) normalized to the number of all identified venom proteins within the analyzed venom sample: (number of proteins of a given family) divided by (number of all identified proteins). The calculation for annotations by MASCOT and PEAKS combined, as well as only-MASCOT and only-PEAKS numbers are listed in Supplementary Table S1.

### 2.3 Gel electrophoresis (1D-SDS-PAGE)

For gel electrophoretic profiling, 2 µg of each venom were dissolved in 12 µl ddH_2_O, mixed with Laemmli-buffer containing 5% 2-mercaptoethanol (v/v) for electrophoresis under reducing conditions or without 2-mercaptoethanol for electrophoresis under non-reducing conditions. Samples were then incubated for 5 min at 95°C. A molecular weight protein marker (Precision Plus Protein All Blue Standard 10–250 kDa, Bio-Rad), as well as venom samples, were loaded on 16.5% Mini-PROTEAN Tris-Tricine gel (Bio-Rad). One dimensional SDS-PAGE was performed for 50 min at 150 V in a Mini-PROTEAN^®^ Tetra Vertical Electrophoresis Cell (Bio-Rad). Coomassie protein staining was performed with ROTI®Blue quick.

### 2.4 Reverse-phase chromatography (RP-HPLC)

For RP-HPLC, 125 µg of each venom was diluted in ddH_2_O + 0.1% TFA (v/v). The chromatographic analyses of venoms were performed on a Dionex ICS-300 SP HPLC system equipped with a preparative C18 column (Vydac 218TP 3 μm, 50 × 4.6 mm) with a constant solvent flow rate of 2 ml/min. The following gradient program was applied (concentrations given in v/v): 100% ddH_2_O + 0.1% TFA for 5 min, MeCN + 0.1% TFA (increasing from 0%–15%) for 3 min, MeCN + 0.1% TFA (increasing from 15%–45%) for 15 min, MeCN + 0.1% TFA (increasing from 45%–70%) for 3 min and MeCN + 0.1% TFA 70% for 4 min. Detection was performed photometrically with a Dionex Ultimate 3,000 Diode Array Detector set to 280 nm and a scan rate of 0.2 s. Process control and data acquisition were done with Chromeleon (version 6.80 SR11 Build 3,160 (183147), Dionex Corporation).

### 2.5 Literature search

To identify relevant publications covering full venom proteome descriptions of the investigated taxa, we referred to the Viperinae Venom Proteomics database by Damm et al. (2021). The reported keyword search until the end of 2020 was extended by an additional investigation from 1 January 2021 to 10 June 2023. To perform the search, we applied the same selection criteria described by Damm et al. (2021), and used the following query: <genus> (Macrovipera) AND <species> (lebetina; lebetinus; razii; schweizeri) AND <subspecies> (cernovi; lebetina; lebetinus; obtusa; schweizeri; transmediterranea; turanica). Extended information of the qualitative proteomic venom comparision in *Macrovipera* is given in Supplementary Table S9.

### 2.6 Cytotoxicity assays

#### 2.6.1 Cell proliferation assays

For all venoms cell-based testing was performed against primary human umbilical vein endothelial cells (HUVECs), Normal Human Dermal Fibroblasts (NHDF), triple-negative breast cancer (MDA-MB-231), and cervix carcinoma (HeLa) cell lines in 96-well plates. Primary HUVECs were isolated from human umbilical cords according to Jaffe et al. (1973). A waiver has been granted for the use of anonymized human material issued by the head of the Research Ethics Committee/Institutional Review Board (REC/IRB) on 15 September 2021 under reference number W1/21Fü. MDA-MB-231 (MDA; ACC-732) cells were purchased from the Leibniz Institute for German Collection of Microorganisms and Cell Cultures (DSMZ, Braunschweig, Germany). NHDF cells were purchased from PELOBiotech (Martinsried, Germany). HeLa cells were a generous gift from Prof. Dr. Rolf Marschalek. MDA-MB231, HeLa, and NHDF cells were cultured in DMEM (PAN-Biotech) containing 10% FCS, 100 U/ml penicillin, and 100 μg/ml streptomycin. All cells were cultured at 37°C in a 5% CO_2_ atmosphere. Primary HUVECs were cultured in collagen G (Biochrom AG, Berlin, Germany)-coated 75 cm^2^ flasks in supplemented endothelial cell growth medium (ECGM) (PELOBiotech, Martinsried, Germany) supplemented with 10% FCS (Biochrom AG), 100 U/ml penicillin, 100 μg/ml streptomycin, 2.5 μg/ml amphotericin B (PAN-Biotech, Aidenbach, Germany), and a supplement mixture (PELOBiotech). All venoms were dissolved in DMSO. For the proliferation assay cells were treated with three different concentrations (0.25, 2.5, 25 μg/ml) of venoms or vehicle (ddH_2_O). HUVECs (2000 cells/well) were seeded into collagen-coated 96-well plates and grown for 24 h. Then, they were treated with venoms. Treated cells were cultured for 72 h, whereas untreated control cells, directly after 24 h, were fixed with a methanol-ethanol (2:1) solution and washed with PBS before they were stained using a crystal violet solution (20% methanol). Similarly, at the end of incubation time, cells treated with venoms were fixed, stained, and unbound crystal violet was removed by washing with distilled water. Finally, cells were left to air dry, and DNA-bound crystal violet was dissolved using an acetic acid solution (20%, Sigma-Aldrich, Steinheim, Germany). Absorbance was measured at 590 nm using a plate reader (Infinite F200Pro, Tecan, Männedorf, Switzerland). The proliferation percentage was calculated by normalizing to the untreated control (24 h) and compared to the water control of 72 h incubation. Similarly, MDA-MB-231 cells (5,000 cells/well), HeLa cells (2,500 cells/well), and NHDF cells (1,500 cells/well) were seeded into 96-well plates, and their proliferation abilities under treatment conditions were tested as described above. Normalized raw-data of the cell proliferation assay is given in Supplementary Table S5.

#### 2.6.2 Cell viability assays

To investigate the cytotoxicity of snake venoms, the embryonal kidney cell line HEK293T and the murine macrophage cell line RAW246.7 were utilized. They were purchased from DSMZ GmbH (Braunschweig, Germany) or ATCC (Virginia, USA), respectively. HEK293T cells were cultured in Dulbecco’s Modified Eagle Medium (DMEM) and RAW264.7 cells in RPMI medium. All media contained 10% fetal calf serum (FCS), and 1% penicillin/streptomycin, and all cells were cultured at 37°C in a 5% CO_2_ atmosphere. To determine cell viability, the OranguTM assay (Cell Guidance Systems Ltd., Cambridge, UK) was performed, as previously described (Ingelfinger et al., 2020). Following this protocol, 2 × 10^5^ RAW246.7 or 2 × 10^5^ HEK293T cells were seeded in 96-well plates. Different concentrations (0, 0.25, 2.5, 25 μg/ml) of snake venoms or vehicle (water) were added. The cells were incubated for 24 h at 37°C and 5% CO_2_. 10 μl of OranguTM cell counting solution was added, incubated for 60 min and absorbance was measured at a wavelength of 450 nm (reference wavelength at 650 nm) at an EnSpire 2,300 Multimode Plate Reader (Perkin Elmer, Lübeck, Germany). To calculate cell viability, the absorbance of vehicle-treated cells was set to 100%, and the snake venom-treated samples were compared to them. Normalized raw-data of the cell viability assay is given in Supplementary Table S6.

### 2.7 Protease activity assays

To assess the protease activity of *Macrovipera* venoms, the protease activity assay kit (Calbiochem) was used following the manufacturer’s manual. Venoms were first resolved in ddH_2_O to a final concentration of 200 μg/ml, 100 μg/ml, and 50 μg/ml. Each concentration was then tested in triplicates against trypsin (1 mg/ml in PBS with 10 mg/ml bovine serum albumin, Calbiochem) and ddH_2_O as controls. In a round bottom 96-well plate, 25 µl fluorescein thiocarbamoyl-casein derivates (FTC-casein), 25 µl Incubation buffer as well as 10 µl of either venom or control were mixed. Incubation was performed at 37°C and 120 rpm for 2 h. Next, 120 µl of trichloroacetic acid (5% in ddH_2_O, CarlRoth) was added to each well and further incubated for 20 min. Then, the plate was centrifuged at 4°C and 500 × *g* for 15 min, before 40 µl of supernatant was carefully transferred to a flat-bottom 96-well plate and mixed with 160 µl Assay buffer. The OD_492_ was measured on a BioTek Eon microplate reader and Gen v2.09. The means of the OD values obtained from each approach were calculated and normalized to the positive (100%) and negative (0%) control. Normalized raw-data of the protease activity assay is given in Supplementary Table S7.

### 2.8 Antimicrobial activity assays

Effects of *Macrovipera* venoms against Gram-positive and negative bacteria were determined on six strains (Table 2). Cryo-conserved cultures were transferred to tryptic soy agar (TSA) plates (Carl Roth) and incubated at 37°C. Single colonies were picked and transferred into either 4 mL Mueller-Hinton II (MH II) medium (BD, Heidelberg, Germany) or in the case of *Listeria monocytogenes* into 4 ml of Brain Heart Infusion (BHI) medium (Carl Roth) and cultivated for 24 h at 37°C and 180 rpm. 4 ml of fresh culture medium were inoculated with each bacterial suspension (*L. monocytogenes* 60 μl, other strains 30 µl) prior incubation for 4 h at the conditions outlined above. A five-step venom dilution series from 2–0.125 mg/ml was created for each venom and tested against all strains using gentamicin (0.02 μg/μl, Sigma-Aldrich, Taufkirchen, Germany) as a positive control. Assays were performed as biological triplicates in 96-multiwell plates with a final volume of 100 µl per well (50 µl of bacterial culture; 50 µl venom solution) after incubation for 48 h at 37°C and 140 rpm. The OD_600_ was measured after 24 h on a BioTek Eon microplate reader and Gen5 v2.09. Normalized raw-data of the antibacterial assay is given in Supplementary Table S8.

**TABLE 2:** Bacterial strains used for testing. Given are the species names of each tested bacterial strain with its respective strain ID. In total seven strains, three gram-negative and four gram-positive, were investigated.

Bacterial species	Gram	Strain ID
*Escherichia coli*	negative	DE3
*Listeria monocytogenes*	positive	20600
*Pseudomonas aeruginosa*	negative	1117
*Pseudomonas aeruginosa*	negative	50071
*Staphylococcus aureus*	positive	2569
*Staphylococcus epidermidis*	positive	35984

## 3 Results and discussion

Despite its medical significance and likely important role in understanding *Macrovipera* venom variations, the venom of *M. schweizeri* has generally received very limited attention, and no proteomic survey has so far been conducted on it. With the present work, we set out to provide the first proteomic insight into the qualitative composition of the Milos viper’s venom.

### 3.1 A first proteomic survey identifies the components of *Macrovipera schweizeri* venom

**FIGURE 2:**
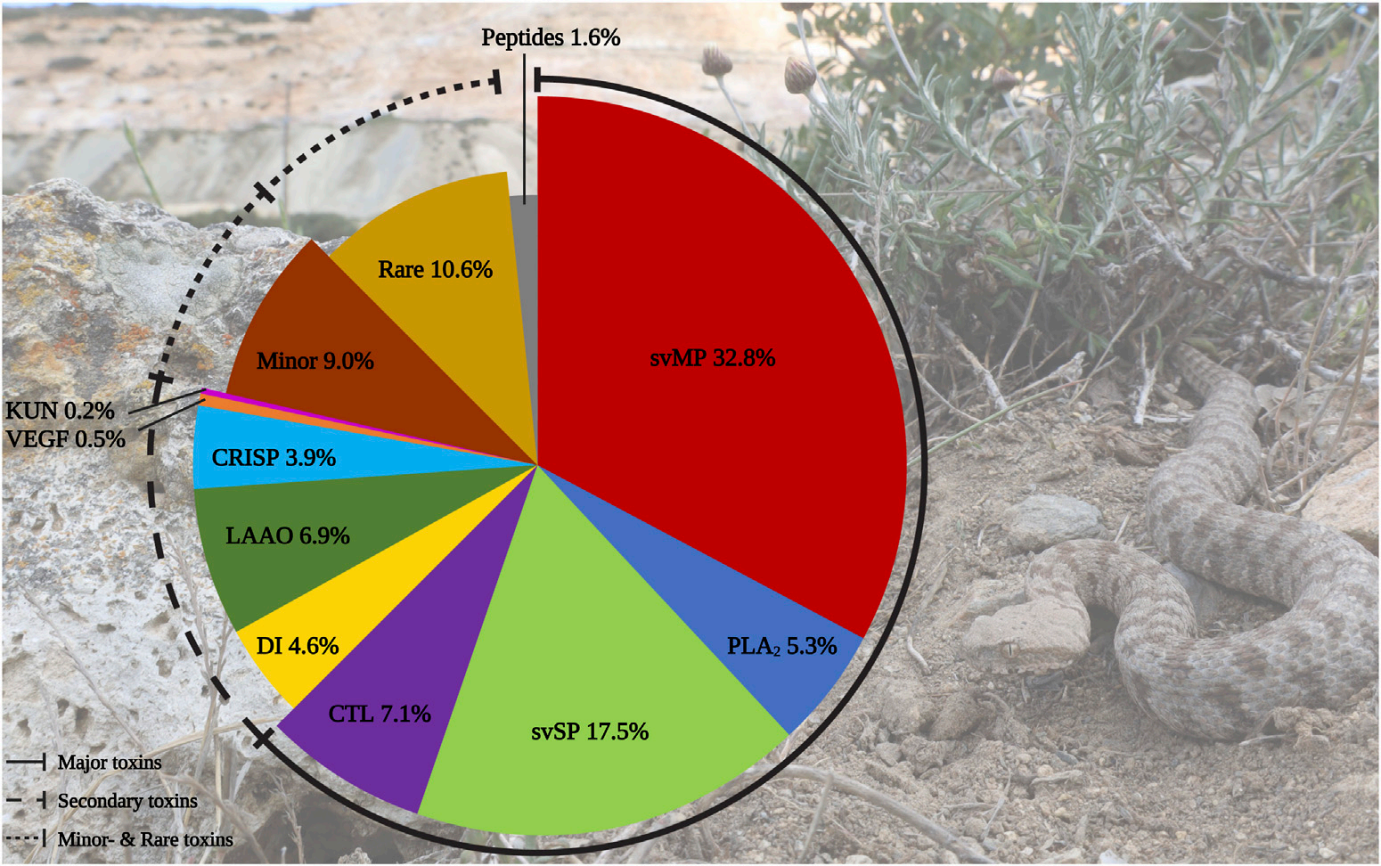
Protein diversity in the Milos viper (*M. schweizeri*) venom proteome. The pie chart illustrates the qualitative venom composition of identified venom proteins within a family in relation to all protein IDs components in percent. The Ttraditional viperine major venom components defined by Damm et al., 2021 (i.e., svMP, svSP, PLA_2_, CTL) constitute the largest fraction of the analyzed venom. Abbreviations: CTL, snake venom C-type lectins and C-type lectin-related proteins (incl. snaclecs); CRISP, cysteine-rich secretory proteins; DI, disintegrins; KUN, Kunitz-type inhibitors; LAAO, L-amino acid oxidase; PLA_2_, phospholipase A_2_; svMP, snake venom metalloproteinase; svSP, snake venom serine protease; VEGF, vascular endothelial growth factors. Photo credits: Thomas Lindner.

In total, we identified 436 components in the *M. schweizeri* venom proteome. While 315 proteins were identified via Mascot, analysis with Peaks recovered 191 proteins. Redundancy between both tools was marginal, and 75 proteins (17.2% of the whole concatenated venom proteome) were shared between both analyses. This indicates that a combination of proteome analysis tools during peptide search may help to identify a larger diversity of venom components, similar to the use of multiple mass spectrometry platforms, enzymatic digestion protocols, and/or multiple assemblers in (proteo)transcriptomic experiments (Lüddecke et al., 2020; von Reumont et al., 2022; Amorim et al., 2023). The venom composition of the Milos viper is illustrated in Figure 2. Tabular data of the concatenated venom proteome, as well as venom compositions retrieved with each tool, are provided in Supplementary Tables S1–S4.

We categorized the identified venom components into the five classes of Old World viper (subfamily Viperinae) venom proteins proposed by Damm et al. (2021), namely major, secondary, minor, and rare venom components, as well as peptides. In line with this classification system, the largest fraction of identified *M. schweizeri* venom protein hits and PSMs is constituted by the major venom components of Viperinae venoms. In general, the venom of *M. schweizeri* seems to follow the general compositional pattern of Viperinae venoms with the majority of identified venom proteins (62.6%) belonging to the classical major viperine venom toxin families. Particularly, the most common proteins were snake venom metalloproteinases (svMP), representing 32.8% of all annotated *M. schweizeri* venom proteins (see Figure 2). This toxin family is related to ADAM proteins (A Disintegrin And Metalloprotease) and subdivided into multiple subfamilies based upon domain architecture: P-I (metalloproteinase domain); P-II (metalloproteinase domain + disintegrin domain); P-III (metalloproteinase domain + disintegrin-like domain + cysteine-rich domain) (Fox and Serrano, 2005; Olaoba et al., 2020). They cause haemorrhage and exhibit strong proteolytic-, coagulopathic, as well as fibrinolytic activities (Ramos and Selistre-de-Araujo, 2006; Gutiérrez et al., 2010). The analyzed *M. schweizeri* venom contains members of all three svMP classes, with P-II and P-III svMP being the most common ones.

The leading toxin identities of all annotated proteins by both search engines are lebetase [Q98995] (P-II svMP) and the coagulation factor X-activating enzyme heavy chain [Q7T046] (P-III svMP), both identified in *M. lebetinus*. Lebetase is a well-known fibrinolytic enzyme from *Macrovipera* venoms, with several isoforms currently described (Siigur et al., 2019). This fibrinolytic svMP is a strong anticoagulant, that affects only α- and β-chains but does not promote plasminogen activation (Siigur and Siigur, 2004). The aforementioned factor X-activating P-III svMP belongs to the VLFXA, and is composed of a svMP heavy chain and two disulfide-bounded C-type lectin-related protein (snaclec) light chains [Q7T045, Q696W1] (Siigur et al., 2001; 2019). This underpins the existence of complete P-IIId svMPs in the *M. schweizeri* venom, an important group of viper svMPs that includes prominent members such as RVV-X from the Russell´s viper (*Daboia russelii*) (Takeya et al., 1992). Besides these two, a plethora of svMPs known from other viperine venoms were observed, including the *M. lebetinus* dimeric and endothelial cell apoptosis inducing VLAIP with both its subunits VLAIP-A [Q4VM08] and VLAIP-B [Q4VM07] annotated, the P-I svMP fibrolase [P83255], and also homologs to the hemorrhagic H3 [R4NNL0] and H4 [V5TBK6] of *Vipera ammodytes ammodytes* (Trummal et al., 2005; Leonardi et al., 2014).

The second most abundant venom protein group is snake venom serine protease (svSP), comprising 17.4% of all identified proteins. These proteolytic enzymes mainly exert coagulotoxicity on several targets via activation of Factor V, Factor X, Prothrombin, Thrombin-like proteins, or through the cleavage of Fibrinogen (Matsui et al., 2000; Kini, 2006). With 70% coverage and 84 assigned peptides inclusive of 58 unique sequences, the most abundant svSP identified is a homolog of the Factor V activator VLFVA [Q9PT41], one the of longest known coagulopathy-inducing toxins of *M. lebetinus* venom (Siigur et al., 2002). Further annotated family members are the svSP-like protein 2 [Q9PT40] and alpha-fibrinogenase [Q9PT40], both described for *M. lebetinus*.

Although less abundant, two additional major Viperinae venom components are present in the *M. schweizeri* proteome produced, namely CTL (C-type lectins and snaclecs; 7.1% of all identified venom proteins) and phospholipase A_2_ (PLA_2_) toxins, to which 5.3% were assigned. The PLA_2_ with the most identified PSMs were the acidic PLA_2_-1 [C3W4R6] and PLA_2_-2 [B6CQR5] from *M. lebetinus*, both members of the D49 subfamily (coverage of 85%). In general, only D49-PLA_2_ has been annotated in *M. schweizeri*. While PLA_2_ have a diverse enzymatic spectrum, mostly myotoxic, neurotoxic or platelet-aggregating effects have been described from snake venom PLA_2_ (Jan et al., 2007). In contrast, C-type lectin-like and lectin-related proteins mostly target clotting factors or cellular receptors (Eble, 2019). They are categorized into homooligomeric sugar-binding snake venom C-type lectins as well as the heterodimeric snaclecs. CTL generally induce an inflammatory response resulting in tissue degradation and edema formation, induce anti-platelet effects and exhibit antibacterial, antifungal and antiparasitic properties (Pathan et al., 2017). Additionally, heterodimeric snaclecs exhibit coagulotoxic effects by binding to clotting factors and platelet receptors (Ogawa et al., 2005). The analysed venom included subunits of the heterodimeric macrovipecetin [C0HKZ6; C0HKZ7] and several snaclecs previously identified from *M. lebetinus* venom.

The secondary toxin families identified comprised five distinct protein groups. Of these, l-amino acid oxidase (LAAO) were the most prevalent ones, constituting 6.9% of all identified venom components. Their functional role in venom is currently unresolved. They are known to catalyze the oxidative deamination of l-amino acids to α-keto acids, with H_2_O_2_ and NH_3_ as side products. They disrupt homeostasis and exhibit various effects, such as cytotoxicity and edema (Lazo et al., 2017; Hiu and Yap, 2020). Antimicrobial and antiviral properties have also been reported (Torres et al., 2010; Paloschi et al., 2018). LAAOs are followed by disintegrins (DI, 4.6%). These often act as competitors for integrin binding, further disrupting homeostasis and inducing apoptosis. Members of this family exhibit strong coagulo-, hemo- and cytotoxic effects, causing tissue damage and haemorrhage (Marcinkiewicz, 2005; Rivas-Mercado and Garza-Ocañas, 2017). The most prominent DI identified within the venom of *M. schweizeri* are the heterodimeric lebeins-1 and -2 [P83253, P83254, Q3BK13], which inhibit the α7β1 integrin binding to laminin-1, with strong myotoxic effects (Eble et al., 2003). VLO5 is another abundant heterodimeric DI, and were identified by its two subunits VLO5A [P0C6A9] and VLO5B [P0C6B0] with up to 96% coverage. VLO5 is known for blocking vascular cell adhesion by α4β1 integrins (Calvete et al., 2003). Obtustatin, a short DI with a KTS-motif known from *Macrovipera* venoms, was annotated with 93% coverage. Also the platelet aggregation inhibiting leberagin-C [C0LZJ5], a D/C-protein, was detected (Limam et al., 2010). The third secondary group were cysteine-rich secretory proteins (CRISP) with 3.9%. The proteomic data indicates the presence of several homologs of crotaline and viperine CRISPs, but no specific *Macrovipera* homolog was detected. The remaining two secondary protein groups contributed a minuscule fraction to the *M. schweizeri* venom diversity. Platelet-derived growth factors/vascular endothelial growth factor (VEGF) proteins constitute 0.5% of the produced *M. schweizeri* venom proteome, and the protease inhibitors from the Kunitz-type (KUN) family the 0.2% of it, with only a single representative, annotated as a homolog to a *Naja nivea* KUN [P00986].

Thirteen protein groups were assigned to the minor and rare viperine venom proteins. Minor components contained 5′nucleotidase (5N, 2.8%), nerve-growth-factor beta (NGF, 2.3%), phospholipase B (PLB, 1.6%), and hyaluronidase (HYAL), plus nucleotide pyrophosphatase/phosphodiesterase (PDE) (both 1.1%). Rare components included 15 snake venom aminopeptidases (AP, 3.4%) mostly of the M1 family and some three-finger toxins (3FTx, 2.8%). The latter are uncommon for vipers and mainly known for elapids, where they often represent the prevalent venom components (Tasoulis and Isbister, 2017; Damm et al., 2021). Eleven 3FTx could be assigned as short-chain cytotoxins with CTx-1 [P01456], −10 [P01453], and −2 [P01463] as the most confident ones. However, at least one identified protein resembled a neurotoxic long-chain 3FTx [P01390] homolog from the Cape cobra (*Naja nivea*). Further rare components included Venom complement activating/C3 homologs (VC3, 1.8%) like the anticomplement protein from *M. lebetinus* described by Gasmi et al. (1994) Acetylcholinesterase (ACE, 0.5%), Lipases (LIP, 0.9%), Glutaminyl-peptide cyclotransferase (QC, 0.7%) for formation of N-terminal pyroglutamate (pE), a common PTM within snake venoms like for bradykinin-potentiating peptides (BPP) and C-type natriuretic peptides (CNP) related peptides, Vespryn (0.5%), and Palmitoyl-protein thioesterase (PPT, 0.2%). Only one group of peptides, belonging to the natriuretic peptides (NP), was identified and represented 1.6% of all venom components. They include a peptide of high similarity to the *Pseudocerastes persicus* natriuretic peptide PNP, that shares 82% identity and 90% similarity to lebetin from *M. lebetinus*, a strong platelet aggregation inhibitor, as well as the svMP-i pEKW and pERW in addition to pEKWPSPK.

Our proteomic assessment revealed a high diversity of protein families in the *M. schweizeri* venom. Overall, the venom of the Milos viper seems to mirror the general composition of viperine venoms, being dominated by typical major viperine venom proteins, such as svMP, svSP and CTL. A large fraction of identified proteins seems to affect the cardiovascular system via coagulotoxic or hemotoxic activities, a mode of action typically displayed by viper venoms (Warrell, 2010; Gutiérrez et al., 2017). Also, a large diversity of identified components (>60% of all proteins), appear to target cellular integrity, and may thus cause tissue damage and/or cytotoxicity. Two protein groups, svMPs and svSPs, comprising together about 50% of the detected venom protein diversity, are known to express strong proteolytic activity (Gutiérrez et al., 2010; Swenson et al., 2021). These first insights into the composition of Milos viper venom are in line with the clinical manifestations elicited by envenomations caused by this species: indeed, bites by *M. schweizeri* are reported to cause lasting pain, swelling, decrease in erythrocytes and heart rate, increase of blood sugar, and hypotension (Cattaneo, 2020). Accordingly, the clinical effects observed following *M. schweizeri* envenoming can be explained by the presented venom proteome. Interestingly, a noteworthy fraction of biomolecules (e.g., LAAO and CTL) are also known to inhibit the growth of bacterial strains, which may only be a secondary natural function to prevent the venom gland from widespread microbial colonization, as recently reported for other toxins from different animal groups (Bocian and Hus, 2020; Lüddecke et al., 2023). In light of this, *M. schweizeri* venom could potentially be worth of consideration in future bioprospecting programs looking for novel anti-infectives

### 3.2 Venoms from *Macrovipera schweizeri* and *Macrovipera lebetinus* subspecies exert similar bioactivities

Our first proteomic survey of Milos viper venom revealed that a large fraction of venom proteins may be involved in coagulotoxic- and cytotoxic activities, exert proteolytic effects, and could also affect prokaryotes. This agrees largely with clinical effects (e.g. hemotoxicicity/coagulotoxicity, organ failure, edema and necrosis) known to occur after envenoming from *M. schweizeri* and *M. lebetinus* (Warrell, 2008; Amr et al., 2020; Cattaneo, 2020; Dehghani et al., 2023). To understand whether our activity predictions based on qualitative proteomic data translate functionally, we tested a subset of bioactivities that we hypothesize to be exerted from *M. schweizeri* venom: We explored the effects of this venom on mammalian cells, investigated its general proteolytic activity, and determined the antimicrobial effects. To better understand the possible differences in venom potency between *M. schweizeri* and closely related taxa, we then compared the activities of the venoms of three *M. lebetinus* subspecies (i.e., *M. l. turanica*, *M. l. obtusa* and *M. l. cernovi*) with those exerted by *M. schweizeri*.

#### 3.2.1 *Macrovipera* venoms are of severe cytotoxicity

A large proportion of *M. schweizeri* venom components are supposedly active on cells and, in agreement with clinical observations, are likely to be causing cytotoxic and tissue-destructive effects. Accordingly, we first examined the effects of our *Macrovipera* panel against mammalian cells.

**FIGURE 3:**
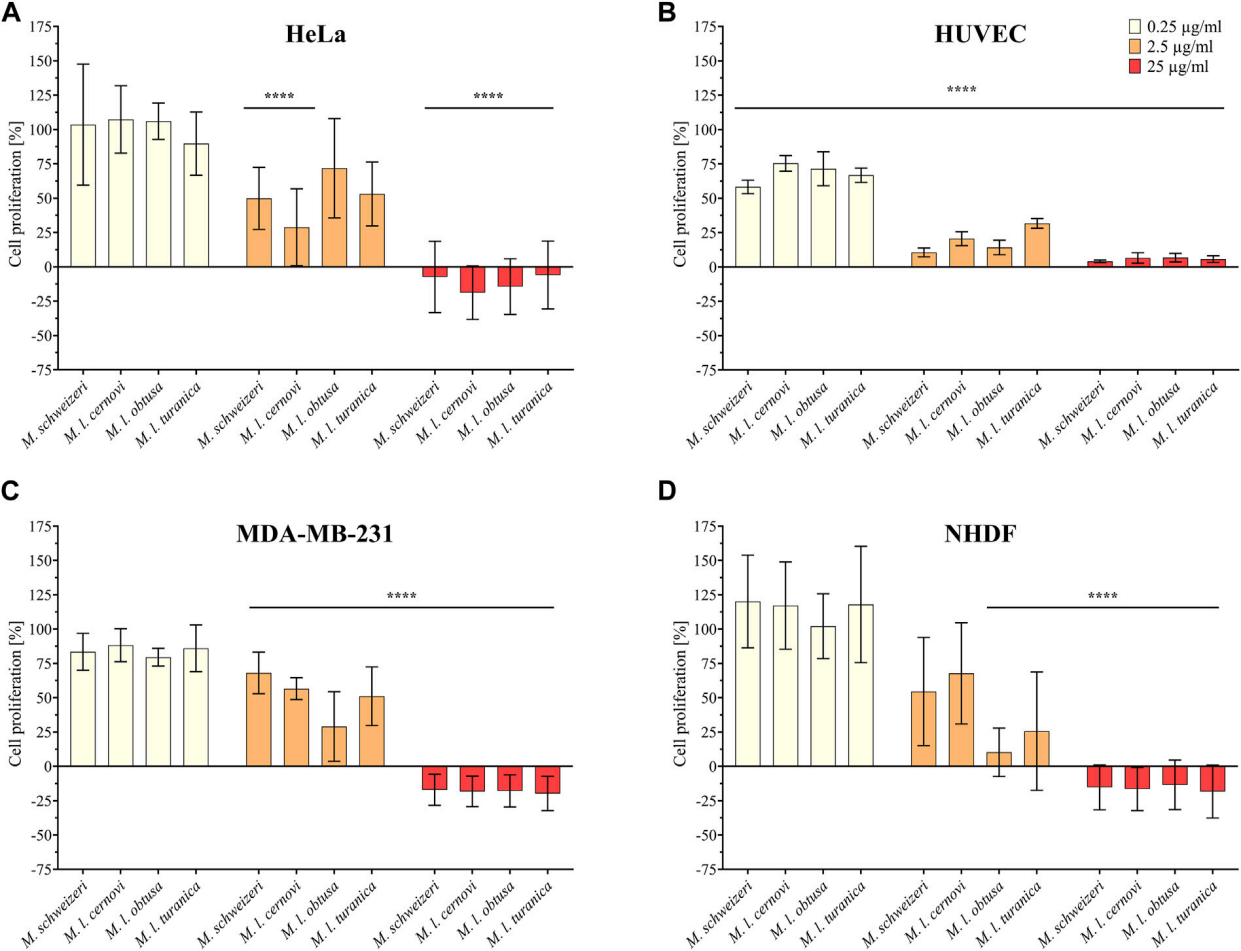
Antiproliferative effects of *Macrovipera* venoms. Given are the results of antiproliferative assays of **(A)** HUVEC, **(B)** NHDF, **(C)** HeLa, and **(D)** MDA-MB-231 cells after exposure to *Macrovipera* venoms at three concentrations (0.25 μg/ml, 2.5 μg/ml and 25 μg/ml). Asterisks indicate significance levels by 2-way ANOVA **(B,C)** or mixed-effects **(A,D)** analysis and Dunnett’s multi comparison test against the control (****: *p* < 0.0001, *n* = 3).

Initially, we examined the antiproliferative effects of *Macrovipera* venoms against four distinct cell types (HUVEC, NHDF, HeLa, and MDA-MB-231). The results of antiproliferative effects are illustrated in Figure 3. We exposed the cells to different concentrations of venom (0.25 μg/ml, 2.5 μg/ml, and 25 μg/ml) and determined the detrimental effects caused. Overall, we found that *Macrovipera* venoms exert potent cytotoxic activities against all cell types tested. All venoms significantly affected cell proliferation upwards from 0.25 μg/ml in primary HUVEC cells. Likewise, all venoms except *M. l. cernovi* significantly affected the proliferation of MDA-MB-231 cells at >2.5 μg/ml. On NHDF cells, significant effects could only be recovered for *M. l. obtusa* and *M. l. turanica* at 2.5 μg/ml. Similarly, no venom was significantly active against HeLa cells when tested at 0.25 μg/ml. At 2.5 μg/ml, only M. schweizeri and M. l. cernovi exhibited a significant effect. In both cell lines, NHDF and HeLa, all macrovipera venoms were significantly active at 25 μg/ml.

**FIGURE 4:**
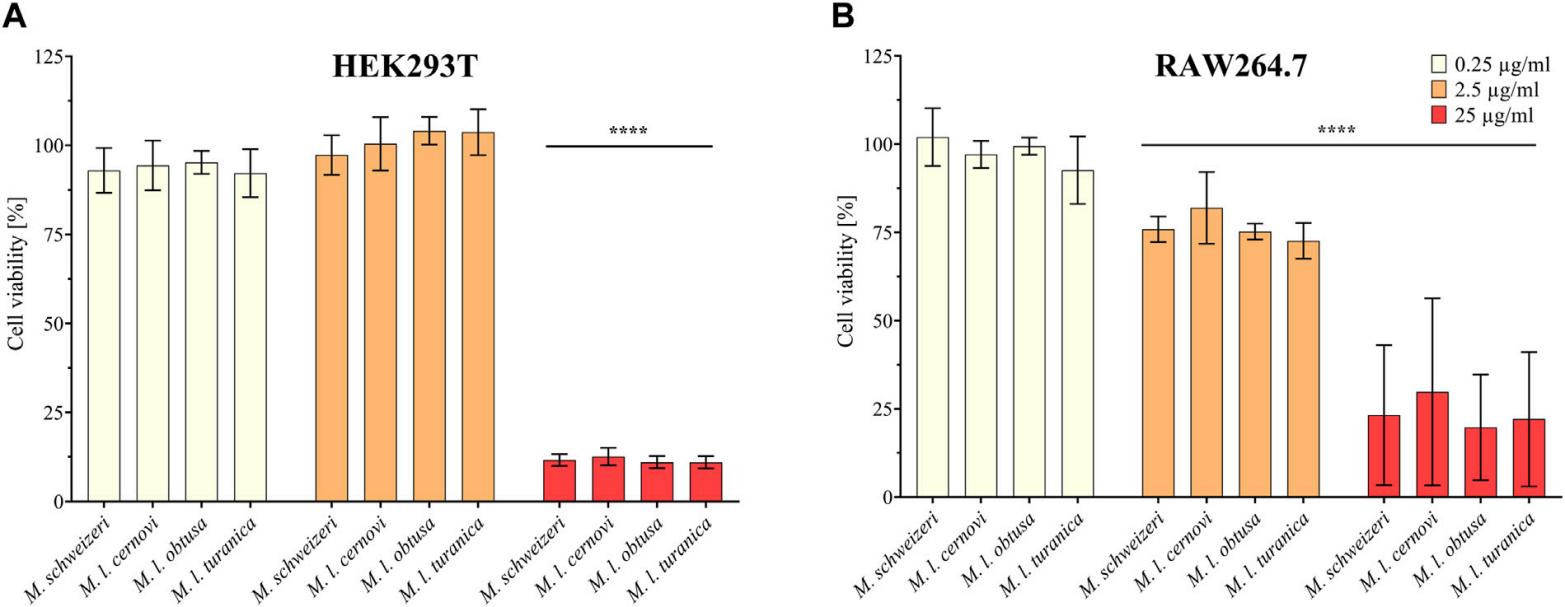
Effects of *Macrovipera* venoms on cell viability assays. Given are the results of cell viability assays of **(A)** HEK293T and **(B)** RAW264.7 cells after exposure to *Macrovipera* venoms at three concentrations (0.25 μg/ml, 2.5 μg/ml and 25 μg/ml). Asterisks indicate significance levels by 2-way ANOVA analysis and Dunnett’s multi comparison test against the control (****: *p* < 0.0001, *n* = 3).

Next, we assessed the cytotoxic effects of *Macrovipera* venoms against HEK293T and RAW264.7 cells via cell viability assays. We employed the same venoms as in the previous antiproliferative assays and used the same concentrations. Results from the cell viability assay are shown in Figure 4. All venoms exerted no effects against HEK293T at 0.25 μg/ml and 2.5 μg/ml but caused a significant effect on cell viability at 25 μg/ml associated with about. 90% reduction of viability in all instances. On RAW264.7 again no effect was detectable at 0.25 μg/μl. However, at2.5 μg/μl, weak yet significant effects were recovered for all venoms that caused a reduction of cell viability towards ca. 75%. Highly significant effects on viability were recovered for all venoms at 25 μg/ml, causing a reduction of cell viability to 25%–30%.

As hypothesized based on our proteomic survey, the cytotoxicity assessment revealed that all venom samples exert severe cytotoxicity against the tested cell lines. In many cases, significant effects were detectable at low (2.5 μg/ml) and sometimes even at very low (0.25 μg/μl) concentrations. Higher concentrations of 25 μg/ml caused significant cytotoxicity of ca. 75%–90% (viability assays) or 90%–100% (antiproliferative assays) on all cells examined. Overall, all *Macrovipera* venoms seemingly display a very similar activity profile against the tested cells, and almost identical activities were measured for HUVEC, MDA-MB-231, HEK293T and RAW264.7 cells. Although some minor variations occur, activity profiles detected on HeLa and NHDF cells also appeared to be relatively similar. Therefore, our data suggest that venoms of *M. schweizeri* and *M. lebetinus* subspecies display very similar cytotoxic properties. This is in agreement with recent studies on the cytotoxicity of *M. l. obtusa* and *M l. lebetinus* venom. These recovered comparable effects on mammalian cell viability at similar concentration ranges as herein detected, thereby confirming our obervations (Nalbantsoy et al., 2012; Ozen et al., 2015; Ozel et al., 2019). This supports the hypothesis that tissue damage associated with envenoming by *M. lebetinus* represents also a potential consequence of *M. schweizeri* envenoming. Such medically relevant effects are exacerbated by the high venom yield of *Macrovipera* snakes. For instance, specimens of *M. lebetinus* have been reported to yield up to 91 mg of dried venom (Latifi, 1984). Although no venom yield data is so far available for *M. schweizeri,* generally smaller than *M. lebetinus* and thus probably producing fewer venom, Milos vipers are likely able to produce still considerable amounts of it. The overall quite similar cytotoxicity data implies that *M. schweizeri* represents a potentially dangerous snake with the capability to cause similar tissue damage as the tested *M. lebetinus* subspecies, and that bites should be considered of medical relevance.

#### 3.2.2 Proteolytic activity of *Macrovipera* venoms

Half of all identified components in the produced Milos viper venom proteome are from the svMP and svSP toxin families. In viperine venoms, including those from previously studied *Macrovipera* spp., such proteolytic enzymes are major drivers of pathogenicity, causing severe coagulo- hemo- and cytotoxicity (Warrell, 2010; Gutiérrez et al., 2017). We thus aimed to determine the extent of protease activity of *M. schweizeri* venom, and to compare it with the venoms of other *Macrovipera* taxa.

**FIGURE 5:**
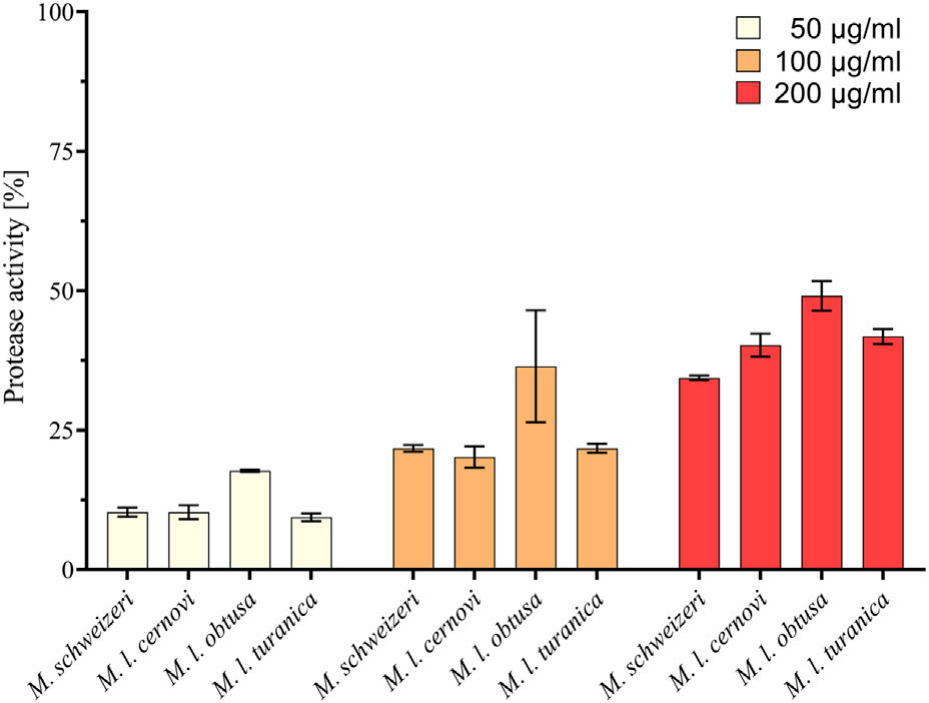
Proteolytic effects of *Macrovipera* venoms. Given is protease activity relative to trypsin as determined via photometry after exposure to *Macrovipera* venoms at three concentrations (50 μg/ml, 100 μg/ml, and 200 μg/ml, *n* = 3) for 2 h.

Our survey of *Macrovipera* venoms revealed that all tested venoms displayed protease activity at all tested concentrations. At a concentration of 200 μg/ml, it ranged from 34.8% to 49.1%, at 100 μg/ml from 20.2% to 36.5% and at a concentration of 50 μg/ml from 9.4% to 17.7% relative to purified Trypsin (see Figure 5). The effects measured were comparable between most taxa investigated, with only *M. l. obtusa* displaying marginally higher activity, especially at 100 μg/ml. At this concentration, *M. l. obtusa* exceeds the other tested taxa by ca. 15% relative protease activity, indicating that differences in quantity or potency of proteolytic enzymes are likely present in this subspecies. However, a bioactivity difference of the detected magnitude certainly is of minor clinical and ecological importance, especially considering that it is only appreciated at lower concentrations. In light of the results of our assays, we conclude that venoms of *Macrovipera* present similar, yet not identical, proteolytic bioactivity patterns, comparable to our observations made on different cell lines.

#### 3.2.3 Venoms of blunt-nosed vipers affect the growth of prokaryotes

Our Milos viper venom proteome unveiled the presence of several protein groups with antibacterial properties. Likewise, antimicrobial activities have already been detected in several viper venoms, including *Macrovipera* (Tõnismägi et al., 2006; Sudharshan and Dhananjaya, 2015; Teodoro et al., 2022). *Macrovipera* antimicrobial activities detected include effects against *Staphylococcus aureus*, *Staphylococcus epidermidis*, *Enterococcus faecalis* and *Enterococcus faecium* (Ozen et al., 2015). We, therefore, implemented antimicrobial activity screenings on six strains from five environmentally- or medically significant prokaryotic taxa, to understand the extent of potential antimicrobial effects in *M. schweizeri* in relation to its congenerics.

**FIGURE 6:**
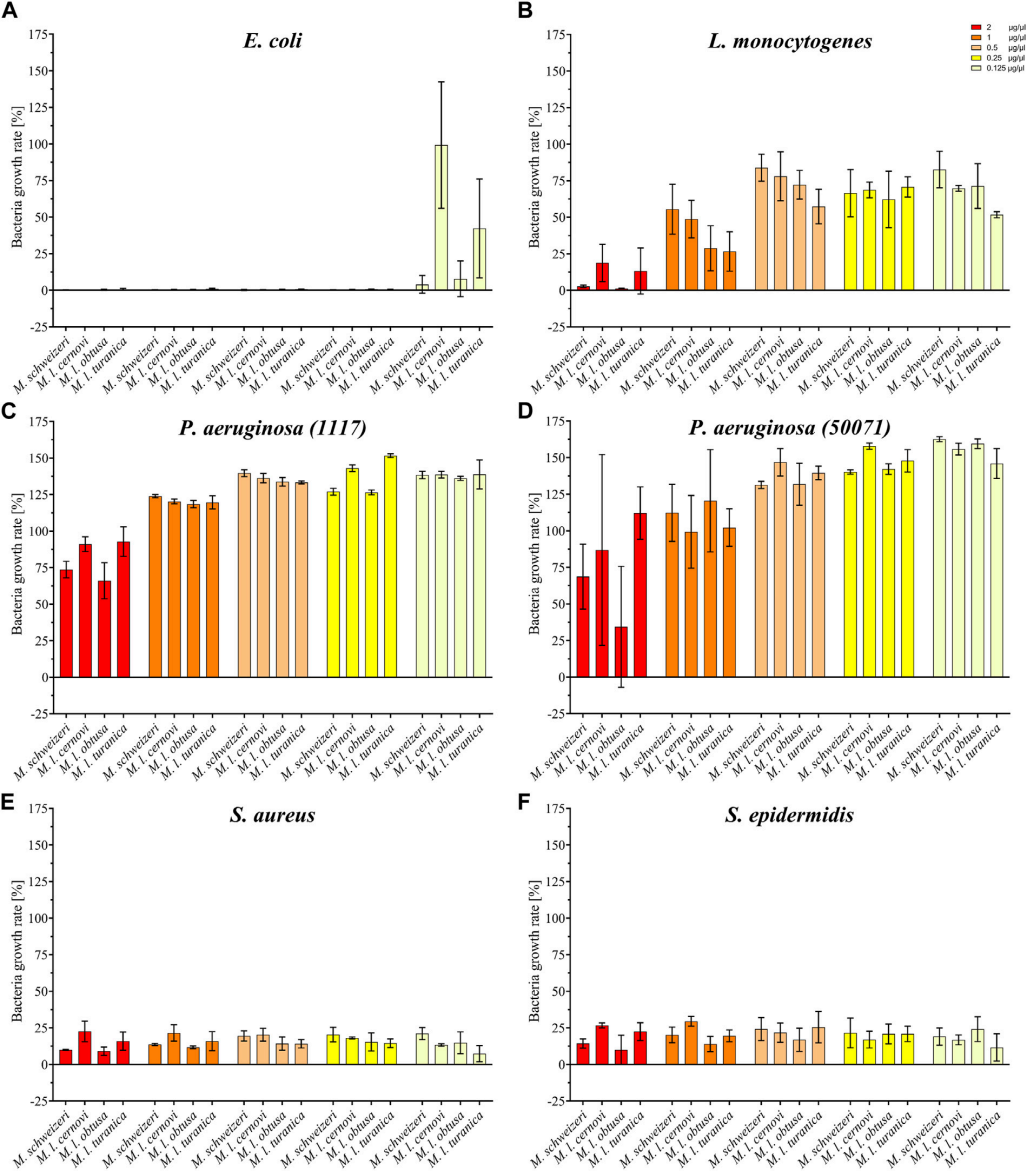
Effects of *Macrovipera* venoms on the growth of **(A)** *Escheria coli*, **(B)** *Listeria monocytogenes*, **(C)** *Pseudomonas aeruginosa* (11179, **(D)** *P. aeruginosa* (50071), **(E)** *Staphylococcus aureus* and **(F)** *S. epidermidis*. Given are the inhibitory effects on bacterial growth determined as OD_600_ and normalized to the gentamycin- (0%) and untreated control (100%) after 24 h exposure to *Macrovipera* venoms at five concentrations (0.125, 0.25, 0.5, 1 and 2 μg/μl, *n* = 3).

Our activity screening revealed that all tested venoms exerted notable antimicrobial effects against several strains (see Figure 6). Already at the low concentration of 0.125 μg/μl, all *Macrovipera* venoms tested inhibited the growth of *S. aureus* and *S. epidermidis*. At the same concentration, growth of *E*. *coli* was heavily reduced by *M. l. cernovi* and *M. l. turanica* venoms, marginally reduced by the venom of *M. schweizeri*, and unaffected by *M. l. obtusa* venom. However, at concentrations exceeding 0.25 μg/μl, all venoms exhibited growth inhibitory effects against *E. coli*. The growth of *L. monocytogenes* was marginally affected by all venoms at concentrations below 1 μg/ml, and heavily affected at concentration 2 μg/ml. The two tested strains of *P*. *aeruginosa* were virtually resistant to *Macrovipera* venom, and a minimal reduction of growth was detected only at a concentration of 2 μg/ml.

Interestingly, similar to our previous bioactivity screens, all *Macrovipera* venoms exhibited a rather similar bioactivity spectrum. The only obvious exception to this case was the absence of activity against *E. coli* displayed by *M. l. obtusa* venom at concentration 0.125 μg/ml. Our data suggests that antimicrobial activity is relatively conserved in *Macrovipera* venoms. Several protein groups were identified in our *M. schweizeri* proteome that can potentially exert antimicrobial activities (e.g., LAAO, CTL), many of which have previously been identified in the venoms of *M. lebetinus* subspecies (Zolfagharian et al., 1998; Ozen et al., 2015). The biological significance of such antimicrobial activities remains unclear. Snake venoms have long been considered to be sterile, but recent studies have shown that complex microbial communities colonize snake venom systems (Esmaeilishirazifard et al., 2022). Accordingly, a potential biological function of such antimicrobial effects could be to control the venom gland microbiome, and to help prevent infection and/or dysbiosis, similar to some arachnid or amphibian toxins (Lüddecke et al., 2018; 2023). However, more research is needed to understand the biological significance of venom-gland-associated bacteria and their interconnection with venom components in snakes.

### 3.3 A comparative view of *Macrovipera* venom compositions

To this end, our proteomic investigation of *M. schweizeri* venom revealed the presence of several protein groups often encountered in other *Macrovipera* venoms (see Damm et al., 2021). A wide variety of identified venom components were found to represent homologs of famous *Macrovipera*-specific toxins, such as lebetase, VLFVA, or macrovipecetin. Moreover, our bioactivity assays conducted against six different mammalian cell lines, six bacterial strains, and one enzymatic activity (protease activity) assay suggested that the general bioactivity pattern of *Macrovipera* venoms on those targets is relatively similar. This led us to hypothesize that the analyzed *Macrovipera* venoms may present overall similar chemical profiles. In order to verify this, we next performed an analysis of *Macrovipera* venoms at our disposal.

#### 3.3.1 Gel electrophoretic and chromatographic profiling suggest similar protein diversity but distinct abundance across *Macrovipera* venoms

For our exploratory analysis of *Macrovipera* venoms, we employed electrophoretic profiling via 1D-SDS-PAGE paired with a chromatographic RP-HPLC profiling. In 1D-SDS-PAGE profiling, venom components are separated per their apparent molecular weight, and venom profiles can be compared based upon their banding patterns (Williams et al., 1988; Gibbs and Mackessy, 2009; Avella et al., 2023). Likewise, the abundance of each retrieved proteinaceous size-group can be estimated by band width and intensity (Menezes et al., 2006). In RP-HPLC profiling, the venom components are separated by their polarity, and the retention times for each measured peak are specific for the eluents. This allows the rough comparison of compound diversity between venoms based upon retention times (Saviola et al., 2015; Zancolli et al., 2017; Avella et al., 2022a), and the area under the curve (measured at a relevant wavelength like 214 nm for peptide bond or 280 nm for aromatic rings), associated to each retention time provides an estimation of the venom component abundances (Calvete et al., 2007).

**FIGURE 7:**
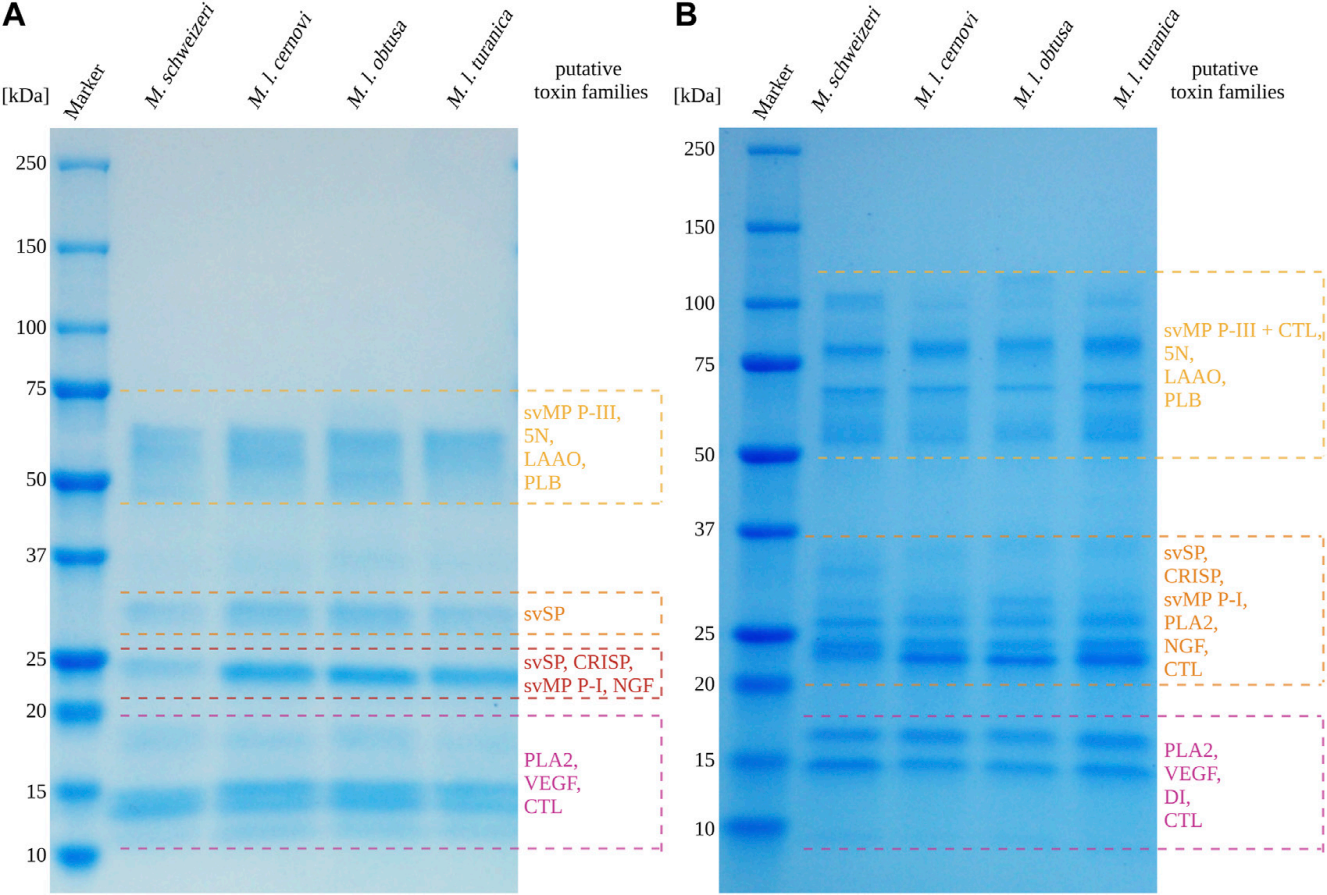
1D-SDS-PAGE of *Macrovipera* whole venoms under **(A)** reduced and **(B)** non-reduced conditions. Shown are the band patterns of examined *Macrovipera* venoms after Coomassie staining with the putative toxin families based on the molecular weight expectation.

Overall, the venoms of the four investigated *Macrovipera* taxa displayedsimilar profiles under reducing and non-reducing conditions, with venom components being visible in different, dominant size groups. Interestingly, several of the detected bands can be used as a rough estimation to groups of diagnostic, toxin-specific bands often retrieved in snake venom SDS-PAGE profiles with a band present: i) between 49 kDa and 65 kDa, matching the diagnostic band size for P-III svMPs, 5N, LAAOs or Phospholipase B; ii) present around 30 kDa, matching the band expected to correspond to svSPs; iii) a group of bands located at about 24 kDa, likely representing CRISPs, svSP, NGF and P-I svMPs. Two additional groups of bands were detected, one around 17 kDa, and the other between 15 kDa and 13 kDa. These bands below 20 kDa may represent lower molecular weight proteins from PLA_2_, CTL and VEGF families. Therefore, the major viperine venom proteins that we identified in our *M. schweizeri* venom proteome seem to be present in all *Macrovipera* venoms. While the overall landscape of *Macrovipera* venom proteins appears relatively consistent in terms of band presence/absence, the abundance of proteins within each group differ, as suggested by band staining intensities. For instance, the proteins at 24 kDa (putative CRISPs) form a very conspicuous band in the *M. lebetinus* subspecies, but are remarkably weaker in *M. schweizeri* (see Figure 7).

**FIGURE 8:**
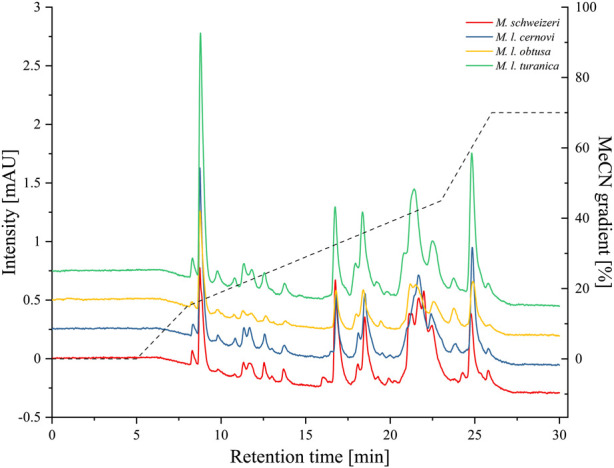
Chromatographic profiling of *Macrovipera* venoms. Shown are the stacked chromatograms of venoms (line graph, offset ≙ 0.5 mAU to previous graph) from *M. schweizeri, M. l. cernovi*, *M. l. obtusa* and *M. l. turanica*, after passage through a C18 reversed-phase column and detection at 280 nm. Dashed graph = Acetonitril (MeCN) gradient.

After electrophoretic profiling, we compared the *Macrovipera* venoms by means of RP-HPLC, and detected eluents at 280 nm per photometry. The venom chromatogram of *M. schweizeri* exhibits an overall chromatographic landscape similar to the other *Macrovipera* venoms considered (see Figure 8). Generally, five major peaks blocks are detectable in all venoms. The first has a retention time of 9 min, the second of 16:45 min, the third of 18:30 min, the broader fourth of 20:45–23 min, and a fifth of 24:50 min. The last two major peaks are characterized by the presence of several shoulders. and a general multipeak pattern. All venoms further exhibit several minor peaks that appear similar between the analysed samples.

#### 3.3.2 Comparison of published proteomes

To further assess the levels of compositional venom variation across *Macrovipera*, we compared our generated *M. schweizeri* dataset and previously published venom proteomes from members of this genus.

**FIGURE 9:**
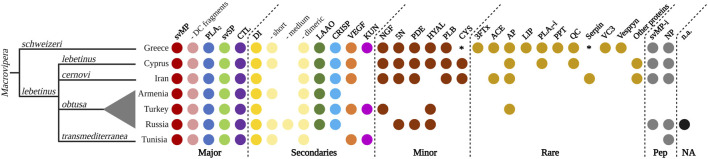
Qualitative venom proteome comparison of seven *Macrovipera* venoms. Shown are the qualitative *Macrovipera* venom proteomes currently described: venoms by full proteome-wide approaches (*M. schweizeri*: this study; *M. l. lebetinus*: Ghezellou et al., 2022; *M. l. cernovi*: Ghezellou et al., 2022; *M. l. transmediterranea*: Bazaa et al., 2005; Makran et al., 2012; *M. l. obtusa* (from three different populations): Sanz et al., 2008, İğci et al., 2012, Pla et al., 2019). Coloured dots indicate the presence of toxin families in each proteome. Asterisks potential components, below our criteria threshold.

Snake venom compositions and their inter- and intraspecific variations received little attention in the past and only grew in interest in recent years (Pla et al., 2019; Zancolli et al., 2019; Mora-Obando et al., 2020; Damm et al., 2021). Investigations developed at these levels helped to elucidate the evolutionary ecology of snake venoms, highlighting the role of certain proteins, protein families and contributing to the medical treatment of snakebite envenomation (Casewell et al., 2020). Our literature search for other proteomics-based venom descriptions across *Macrovipera* yielded six previously published works, exclusively covering *M. lebetinus* and its subspecies (see Figure 9). The proteomic assessment of *M. schweizeri* presented in this study paves the way to the development of a first interspecific comparison of venom profiles within the genus *Macrovipera*, and provides insight into the conundrum of interspecific venom variation in this medically relevant snake genus.

Our comparison of proteomic datasets shows that the four major viperine venom components (i.e., svMP, PLA_2_, svSP and CTL; *sensu* Damm et al., 2021) are present in all *Macrovipera* venoms analysed to date. Particularly, svMPs appear to be the prevalent toxins, with several members from the P-I, P-II and P-III groups, but also with similarity with D/C fragments such as leberagin-c. Among the secondary toxins, DI are found in all investigated venoms. More specifically, a variety of abundant dimeric DI, such as VLO4, VLO5 or lebein, were detected. Interestingly, the types of recovered DIs differed between the examined taxa. Short DI were not found in the venoms of *M. l. cernovi* and the Turkish *M. l. obtusa*, while they were identified in all other venom samples, where they were also shown to carry the diagnostic KTS-motif. Medium DIs are only found in Turkish *M. l. obtusa* venom. LAAO and CRISP are found in every investigated *Macrovipera* venoms, except *M. l. transmediterranea.* VEGF and KUN are not found in the venom of *M. l. obtusa* from Armenia and Russia, and KUN are also lacking in *M. l. lebetinus* and *M. l. cernovi*. This indicates that KUN only features a rare or low abundant protease inhibitor in *Macrovipera* venoms. Considering the minor and rare toxins, as well as peptides, the most recent proteomic studies of *M. schweizeri, M. l. lebetinus* and *M. l. cernovi* show a more extensive detection of those. This could be an effect caused by the technology (e.g., shotgun) applied in these studies. Less abundant proteins are often better detectable in whole venom digestions, than in multi-step workflows, such as a combination of HPLC columns and densitometric detections in SDS gels, that might act as a filter. The minor toxins NGF, 5N, PDE, HYAL, PLB and cystatin (CYS) are found in the venoms of *M. schweizeri, M. l. lebetinus* and *M. l. cernovi*, except for HYAL (absent in *M. l. cernovi*) and CYS (below the criteria threshold in *M. schweizeri*). For the other venoms, only few minor toxin families are identified from the Russian and Turkish *M. l. obtusa* proteomes. It is worth noticing that in the two oldest datasets analysed herein (i.e., Armenian *M. l. obtusa* from Sanz et al., 2008, and *M. l. transmediterranea* from Bazaa et al., 2005), no minor or rare toxins could be detected. As for peptides, svMP-i were revealed within all *Macrovipera* except *M. l. transmediterranea*. Additionally, venoms of all taxa were found to harbour NPs (although inconsistently), and several peptides that could not be annotated were present in all Russian *M. l. obtusa*. In the venoms of *M. l. obtusa* from Armenia and Turkey, no venom peptides were described. Over all, the proteomic investigation of *M. schweizeri* presents the most complex venom composition, with unique venom families firstly observed in *Macrovipera,* such as 3FTx, LIP, PPT, VC3 and Vespryn. In general, the investigated *Macrovipera* venoms appear to be very similar in terms of major and secondary toxins. However, differences are suggested to occur only on the level of minor and rare toxin families that constitute only a minuscule fraction of the overall toxin diversity in viperine venoms.

## 4 Conclusion and future perspectives

Vipers of the genus *Macrovipera* are widely distributed across the Palearctic, where they inhabit very diverse habitats. The different populations and species that dwell in distinct habitats are likely exposed to different local selective pressures, possibly related to trophic factors such as different prey types, abundances, and/or susceptibility to venom. The Milos viper, for instance, is known to primarily hunt avian prey, in contrast to other *Macrovipera* species that are known to mainly feed on small mammals. Therefore, considering that diet has repeatedly been shown to be an important driver of variation in snake venom, if distinct members of the genus *Macrovipera* present different feeding ecologies, it is reasonable to assume that they could feature divergent venom profiles. In light of this, one of the major goals of our study was to use our novel *M. schweizeri* venom proteome as a baseline to better understand the potential extent of venom variation in blunt-nosed vipers.

Interestingly, our analyses consistently showed that the venom profiles of all examined species were relatively similar, as indicated by chromatographic- and electrophoretic profiling. Likewise, the results of the bioactivity profiling aligned with these findings, as exerted bioactivities were comparable in almost all cases. When looking at previously published proteomes in tandem with our novel *M. schweizeri* proteome, we recognized that also the proteomic venom profiles are relatively similar across *Macrovipera*. The major and secondary viperine venom components are present in comparable numbers, and represent the largest venom fraction within each proteomic profile. For instance, in our *M. schweizeri* dataset major and secondary components represent ca. 80% of all identified venom components. The only apparent exception to this is the venom of *M. l. transmediterranea*. When exploring at its published proteome, we found that it differs profoundly from its congenerics. For instance, its venom seems to be mostly composed by svMP, with levels of PLA_2_ and svSP being dramatically lower than other *Macrovipera* taxa. Additionally, it lacks LAAOs and CRISPs, and appears to be more similar to venoms from some viper species of the genus *Daboia* (Momic et al., 2011; Makran et al., 2012). Interestingly, the relationship between *Macrovipera* and *Daboia* has been subject to heated taxonomic discussions in the past, and for several years the Moorish viper *Daboia mauritanica* was considered a member of the genus *Macrovipera* (see Herrmann et al., 1992). Nonetheless, *Macrovipera* and *Daboia* are currently recognised as two distinct genera, as strongly supported by molecular data (see Lenk et al., 2001; Wüster et al., 2008). It should be pointed out that *M. l. transmediterranea* was described by Nilson and Andrén (1988) on the basis of old museum specimens collected in non-specified Algerian and Tunisian localities, and is nowadays generally considered of dubious validity (see Sindaco et al., 2013; Aghasyan et al., 2021). The seemingly divergent venom profile of *M. l. transmediterranea* produced by Bazaa et al. (2005), together with the unspecified origin of the venom samples analysed, could thus indicate that the specimen from which the venom samples were taken might have been a misidentified Moorish viper (*Daboia mauritanica*). Future taxonomic studies and herpetological surveys should be performed to resolve the taxonomic status of *M. l. transmediterranea* before this taxon can be properly discussed and compared with other *Macrovipera* taxa for its venom. Facing our acquired data and interpreting them in light of previously published works, we conclude that the venom profiles of *Macrovipera* spp. (with the exception of *M. l. transmediterranea*) are overall similar, with the same important toxin families being present in all of them. This aligns also with the clinical effects observed after *Macrovipera* envenoming, and is further supported by the overall high efficacy of Inoserp European viper antivenom against *Macrovipera* venoms (Chowdhury et al., 2021a). While the qualitative evaluation of the examined venoms did not yield many differences between the tested taxa, some quantitative differences may be present. Indeed, in our chromatographic and electrophoretic profiling, differences in peak areas and band intensities are clearly present, indicating that although the same components are present in the examined venoms, these may occur in different amounts. Such quantitative differences would explain the few different effects measured in our bioactivity profiling (e.g., the somewhat higher protease activity of *M. l. obtusa*).

Although the assessment presented here improves our understanding of *Macrovipera* venoms, there are some constraints that must be taken into account when interpreting our data. The first important issue is that the species *M. razii* has so far not been investigated for its venom. Accordingly, our interpretations are intrinsically biased to only a subset of taxa within *Macrovipera*, and it is certain that the future inclusion of *M. razii* will be crucial to fully elucidate the presence of intrageneric differences in venom profiles within *Macrovipera*. Another impotant consideration in this context is, that venom variation in *Macrovipera* may occur in dependence to local adaption. In species with large distribution ranges, such as most taxa within the blunt-nosed vipers, it is important to recognize this factor. We therefore recommend, that future studies should include more specimen from distinct habitats (e.g. *M. schweizeri* from different islands of the Kyklades) to account for this factor. It is also interesting, that despite we unveiled a relatively similar venom profile, distinct bioactivities on prey-specific targets were already measured (Chowdhury et al., 2021a; Chowdhury et al., 2021b). This could be explained by selective pressures acting on distinct sites on the proteins leading to increased target specificity and potency. In order to better understand the evolution of toxins within the blunt-nosed vipers, it would be important to erect a sequence collection either by traditional approaches (e.g. toxin isolation followed by Edman degradation) or modern approaches (e.g. venom gland transcriptomes or species genomes). We consider it an important task to perform such experiments and are confident, that they will pave a way towards a better understanding of the evolutionary driving forces behind *Macrovipera* venoms. Lastly, it is also important to take into account that the *Macrovipera* venoms we compared were analysed through different proteomic technologies and in different labs. Each proteomic platform is affected by different experimental constraints, with affects comparability. In order to reliably unveil differences in venom compositions between *Macrovipera* taxa, it is therefore of pivotal importance that future studies are performed on an unified system. Additionally, in order to yield maximum accuracy while also allowing to assess quantitative differences, the combined application of bottom-up and top-down proteomics approaches to yield maximum accuracy while also allowing to assess quantitative differences, including also the free accessibility of raw data for reanalysis (Damm et al., 2021), would be ideal. Such integrative studies across the genus *Macrovipera* would be of highest significance for basic research on snake venom ecology, and would also provide useful tools to battle snakebite caused by this clade of medically relevant Eurasian vipers.

## Data Availability

The datasets presented in this study can be found in online repositories. The names of the repository/repositories and accession number(s) can be found in the article/Supplementary Material.
